# Introducing a portable electrochemical biosensor for *Mycobacterium avium* subsp. *paratuberculosis* detection using graphene oxide and chitosan

**DOI:** 10.1038/s41598-023-50706-z

**Published:** 2024-01-02

**Authors:** Nahid Naghshgar, Saied Hosseinzadeh, Abdollah Derakhshandeh, Ruhollah Shaali, Mohammad Mahdi Doroodmand

**Affiliations:** 1 Department of Pathobiology, School of Veterinary Medicine, Shiraz University, Shiraz, Iran; 2 Department of Food Hygiene and Public Health, School of Veterinary Medicine, Shiraz University, Shiraz, Iran; 3 Department of Chemistry, College of Science, Shiraz University, Shiraz, 71454 Iran

**Keywords:** Biotechnology, Chemical biology, Nanoscience and technology

## Abstract

In this contribution, a novel, low-cost, high throughput, and ultra-selective electrochemical DNA nanobiosensor was developed for accurate on-site detection of *Mycobacterium avium* subspecies *paratuberculosis* (MAP) in real media for practical diagnosis of Johne's disease (JD). The method was designed based on the immobilization of graphene oxide and chitosan biopolymer on the surface of a glassy carbon electrode, modified by electrochemical immobilization of graphene oxide and chitosan biopolymer, followed by activation of biopolymer via 1-ethyl-3-(3-dimethylaminopropyl) carbodiimide hydrochloride and N-hydroxy succinimide (EDC/NHS) coupling system. Afterward, the commercial probe DNA (ssDNA) was stabilized on the activated electrode surface to prepare an ultra-selective ssDNA-stabilized nanobiosensor for MAP sensing called “ssDNA-stabilized GO-CH-EDC/NHS-modified electrode”. Several characterization methods distinguished the bioelectrode. The DNA hybridization between the nanobiosensor and target DNA was confirmed by cyclic voltammetry and differential pulse voltammetry. "At optimal experimental conditions, the nanobiosensor showed a linear range of 1.0 × 10^−15^–1.0 × 10^−12^ mol L^−1^, a detection limit as low as 1.53 × 10^−13^ mol L^−1^, and a repeatability with a relative standard deviation (%RSD) of 4.7%. The reproducibility was also appropriate, with a %RSD of about 10%. It was used to diagnose MAP in real samples with highly accurate results. Therefore, the developed nanobiosensor can be used for clinical diagnosis of MAP.

## Introduction

One of the most common cattle (domestic animals) bacterial diseases is called Johne’s disease (JD), caused by infection of cattle with *Mycobacterium avium* subsp. *paratuberculosis* (MAP)^1,2^. From an economic perspective, due to reducing the milk production from the infected animals with MAP, about 1.5 billion dollars were annually loosed only from US dairy industries^3^. Besides, emaciation and the necessary culling of cattle infected with MAP have been calculated to cost about 250 million dollars per year for the dairy industries of Canada^4^. Considering the above economic severe issues, the accurate, precise, and rapid point-of-care detection of MAP for the diagnosis of JD seems to be substantial from both economical and clinical points of view^5–7^.

More importantly, there is some evidence for the association of MAP with Crohn's disease in humans, making its accurate detection more critical^8,9^. There are several laboratory-based clinical tests for the diagnosis of JD including enzyme-linked immunosorbent assay (ELISA) and polymerase chain reaction (PCR), with result’s accuracy of (70–90%) for identification of antibody of MAP in cattle and qualification of DNA of MAP into the collocated samples, in order^4,10,11^. Besides, bacterial culture analysis has also been used toward JD diagnostic aims^3,12^. Despite the significant advantages of current methods, for instance, accurate and precise detection of MAP, the current methods often suffer several serious drawbacks, including specialized training, expensive equipment, and qualitative responses^3,4^. Moreover, the bacterial culture analytical method is very time-consuming (requiring 7–12 weeks for diagnosis)^3,13^.

From another point of view, the PCR-based methods (e.g., nested PCR, single PCR, and real-time PCR) are frequently unsuitable for rapid point-of-care detection due to their requirement for thermal cycling and long amplification time^4^. Furthermore, the accessibility of these diagnostic clinical tests toward the on-site diagnosis of JD is a limitation for several developing countries. Due to the above drawbacks, the current methods are not applicable for the on-site point-of-care detection of MAP and rapid diagnosis of JD, which leading to problems in the effective control of JD. Hence, the design and development of rapid, cost-effective, accurate, precise, on-site point-of-care methods for qualification and quantification of MAP are necessary for controlling the JD in its initial steps. Recently, the fast development of nanotechnology and materials sciences has led to the synthesis and development of high throughput nanomaterials with excellent catalytic^14–16^, optical^17^, and biomedical properties^18^ with respect to different applications in nano/biocatalysis^19^, enzyme immobilization^20–22^, and nano/biosensor development^23,24^. Among the various applications of nanomaterials, their applications in biosensing pathogens (e.g., viruses and bacteria), proteins, and small molecules, as well as disease diagnosis, attracted the focus of researchers due to their importance for clinical, industrial, and economic considerations^25,26^. Among different nano/biosensing assays, the electrochemical biosensors are more suitable for on-site point-of-care diagnosis of diseases for their rapid and accurate responses and precise inexpensive and miniaturized equipment^3^.

In contrast to various electrochemical nanobiosensors, the electrochemical DNA-based nanobiosensors have been widely used for various analytes such as nucleic acids, proteins, and pathogens, as well as for clinical disease diagnosis. This is due to their disability, rigidity, stability, reproducibility, specificity, and high sensitivity^27^. Regarding the MAP biosensing, the current biosensors reveal several serious disadvantages, including low sensitivity, poor selectivity, and low detection range^28–30^. Therefore, the current biosensors are not applicable for point-of-care detection of MAP, consequently the development of a rapid, sensitive, and selective biosensor for MAP quantification in real media is necessary.

Hence, in this contribution, a novel, low-cost, high throughput, highly sensitive, and ultra-selective electrochemical DNA nanobiosensor was developed to detect MAP in real media for practical diagnosis of JD. Briefly, the method was designed based on the immobilization of graphene oxide and chitosan biopolymer on the surface of a glassy carbon electrode during, modification with electrochemical immobilization of graphene oxide and chitosan biopolymer, followed by activation of biopolymer via EDC/NHS coupling system. Afterward, the commercial probe DNA was stabilized on the activated electrode surface to prepare an ultra-selective nanobiosensor for MAP sensing purpose. Then, the electrode was characterized by several characterization methods. The DNA hybridization between the nanobiosensor and target DNA was confirmed by cyclic voltammetry and differential pulse voltammetry. The effective parameters were optimized, and the figures of merit of the introduced nanobiosensor, including linear range, the limit of detection, repeatability, reproducibility, and selectivity, were investigated. Finally, it was employed for qualitative and quantitative analysis of real samples toward the practical diagnosis of JD.

## Results and discussion

### Characterization of graphene oxide nanoparticles (GO)

Initially, the as-prepared graphene oxide nanoparticles were characterized by imaging method using Scanning Electron Microscopy (SEM) (TESCAN-Vega3 Brno, Czech Republic). In this regard, the SEM image of the as-prepared graphene oxide nanoparticles was recorded (Figure s1). The results revealed that the prepared graphene oxide nanoparticles have a size distribution of over 80–215 nm with an average size of 160 nm.

In addition, the crystalline properties of the as-prepared GO were evaluated by patterned X-ray diffraction (XRD) d8 advance, Bruker, Germany analysis. As shown in Figure s2, an abroad band centered at 2θ of 13.5° and a sharp peak at 41.2° strongly revealed the amorphous crystalline structure of the as-synthesized GO, which were in good agreement with previously reported in the litreture^21^.

### Characterization of GO-CHI-EDC/NHS modified electrode

#### Morphological properties

**Figure 1:**
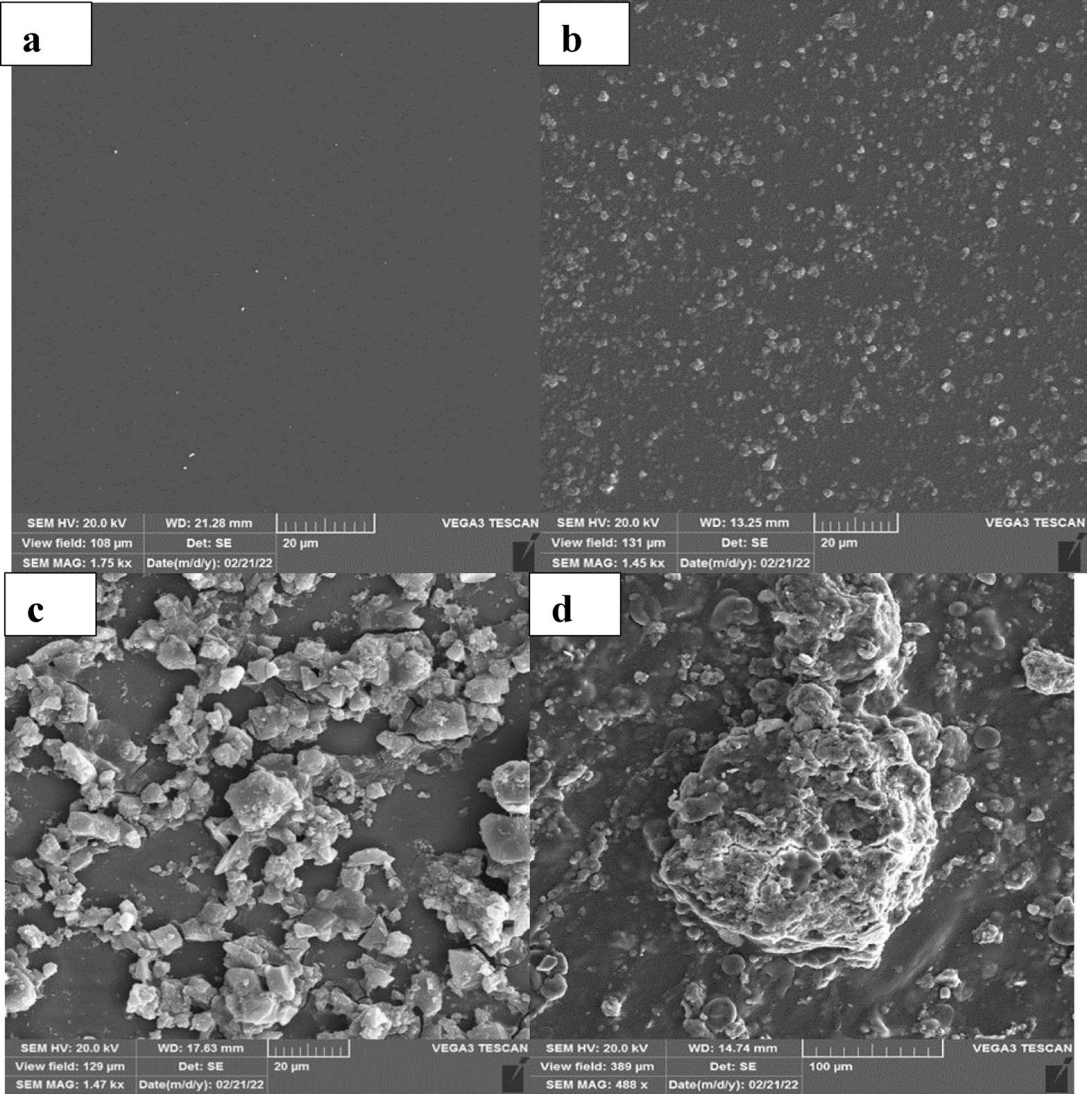
SEM images (**a**) bare GCE, (**b**) EDC/NHS modified GCE, (**c**) GO-CH-EDC/NHS modified GCE and (**d**) ssDNA-stabilized GO-CHI-EDC/NHS modified bioelectrode.

The morphology of the fabricated bioelectrode was investigated by Scanning Electron Microscopy (SEM) images). In this regard, the SEM images of bare glassy carbon electrode (GCE) (Fig. 1a), EDC/NHS modified GCE (Fig. 1b), GO-CH-EDC/NHS modified GCE (Fig. 1c), and ssDNA-stabilized GO-CH-EDC/NHS modified bioelectrode (Fig. 1d) were recorded. The results showed that there were no particles on the surface of the bare (unmodified) GCE, while after its modification with EDC/NHS, the successful deposition of EDC/NHS particles was directly proved via visualization by SEM image, as shown in Fig. 1b. Besides, the SEM image of the GO-CH-EDC/NHS modified GCE (Fig. 1c) revealed the uniform deposition of GO-CH composite on the surface of the electrode. The immobilization of ssDNA on the surface of the GO-CH-EDC/NHS modified GCE was also proved by the SEM imaging, which showed the presence of ssDNA on the surface of the electrode after the immobilization process, as can be seen in Fig. 1d.

#### FT-IR analysis

For an illustration of the successful bonding of the different species on the surface of the GCE electrode, middle -Fourier-transform infrared spectroscopy (FT-IR) (Tensor II, Bruker Company ,Germany) (in the frequency range of 400–4000 cm^−1^) was utilized as the primary analysis (Figure s3). The results showed that after several oxidation–reduction cycles, many oxidation moieties were constructed, and the electrode surface was prepared for immobilization/deposition of different components of the proposed biosensor. Regarding GCE, the strong peak positioned at 3335 cm^−1^ frequency in the FT-IR spectrum is assigned to the presence of –OH moieties on the electrode surface. Besides, the peaks positioned at frequencies of 1562, 1645, and 2972 cm^−1^ were related to the functional groups of NHS, proving its deposition on the electrode surface (spectrum a). In addition, the peaks at 2703, 1264, and, 1129 cm^−1^ can be used to deduct the presence of EDC/NHS on the surface of the electrode during activation of –COOH and –OH functional groups. After the activation reaction, the O–H stretching in the FT-IR spectrum was disappeared, revealing the successful bonding of EDC/NHS with the electrode surface. After modification of the electrode with GO-CH nanocomposite, some new peaks are observed in the FT-IR spectrum, which was related to the aromatic structure (frequency: 1596 cm^−1^), aldehyde moieties (Frequencies: 1724 and 2886 cm^−1^) as well as 2900 for C-H stretching of the GO compound (sample c). Moreover, the FT-IR of the ssDNA-stabilized GO-CH-EDC/NHS modified bioelectrode revealed the appearance of the vibrational peaks of alkene (2361 cm^−1^), aldehyde (2872 cm^−1^), and the hydroxylic moiety of carboxylic acid in (3356 cm^−1^) (See sample B) which are related to the chitosan.

#### EDX analysis

Another test used to detect components attached to the surface of the electrode was the EDX, which provided significant results. The Energy-dispersive X-ray spectroscopy (EDX) (Shimadzo, Japan). Effects of the bare GCE, EDC/NHS modified GCE, GO-CH-EDC/NHS modified GCE, and ssDNA-stabilized GO-CH-EDC/NHS modified bio electrode were represented in Table s1. The results revealed that, the oxidation step was performed successfully as the oxygen claim was increased significantly. On the other hand, after bonding steps, the nitrogen claim increase was deducted as a successive period. Based on these considerations, it can be concluded that both EDC/NHS and chitosan biopolymer were successfully bonded on the surface of the GCE. Besides, the increase of carbon percentage after electrode modification with GO-CH nanocomposite proved the presence of the nanocomposite as mentioned earlier on the surface of GCE. Besides, the ssDNA binding with the modified electrode can be confirmed by EDX due to the nitrogen claim growing.

#### Electrochemical behavior

**Figure 2:**
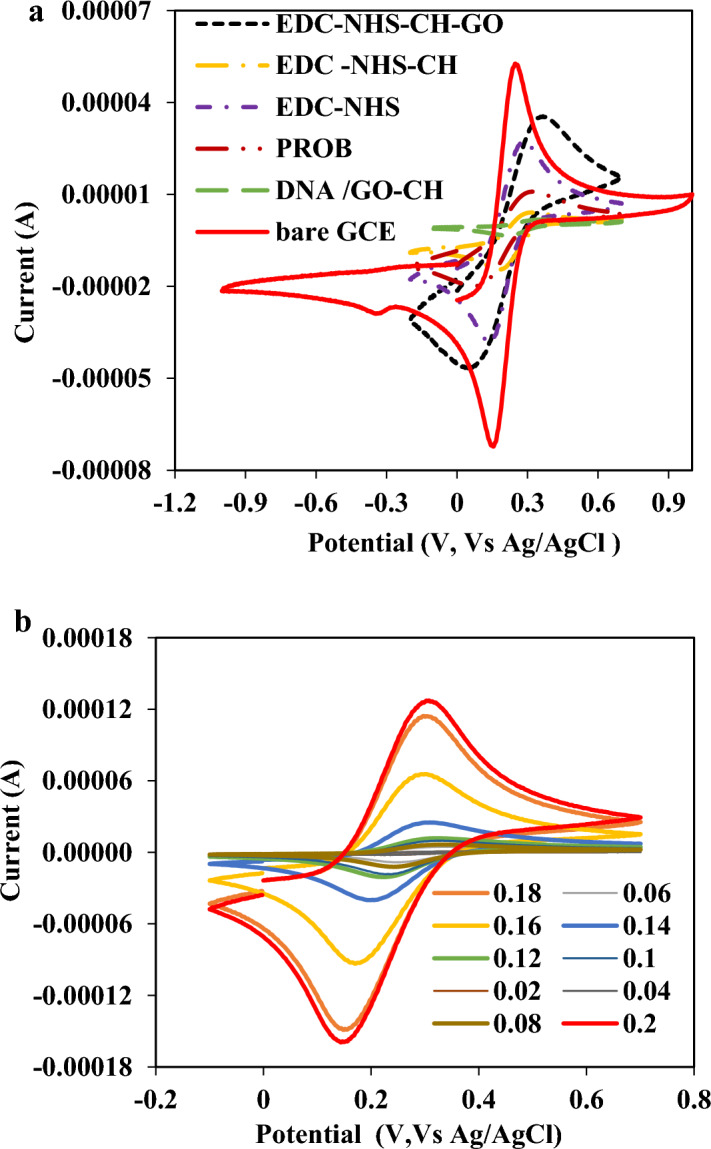
(**a**) Cyclic voltammograms of bare GCE, EDC/NHS modified GCE, GO-CH-EDC/NHS modified GCE, and ssDNA-stabilized GO-CH-EDC/NHS modified bioelectrode at a scan rate of 100.0 mV s^−1^ and (**b**) Cyclic voltammograms of the bioelectrode in Zobel’s solution at different scan rates 0.02–0.2 V s^−1^.

Both cyclic voltammetry (CV) and differential pulse voltammetry (DPV) were performed to evaluate the interface properties and electrochemical behavior of the modified electrodes. The measurements were carried out in 0.001 mol L^−1^ ferro-/ferricyanide (Fe (CN)_6_^(4−/3−)^ containing 0.1 mol L^−1^ KCl between − 1.0 and 1.0 V (vs. Ag/AgCl) at a scan rate of 100.0 mV s^−1^. As shown in Fig. 2a, when the EDC/NHS-modified GCE was modified by binding of GO-CH nanocomposite, an increase in the anodic potential and a decrease in the reduction potential of the electrode was observed because the GO could enhance the effective surface area and the conductivity of the electrode. In contrast, after immobilization of the ssDNA probe on the surface of the GO-CHEDC/NHS-modified GCE, the ssDNA blocked the electron transfer, and the peak current decreased probably due to the electrostatic repulsion between a negatively charged electrolyte solution and the ssDNA. Notably, a further decrease in the peak current can be observed when the target DNA solution was dropped onto the electrode surface, indicating that the capture probe was successfully hybridized with the oligonucleotides of the target DNA. Moreover, the CVs were recorded at different scan rates to illustrate the electroactivity of the as-prepared bioelectrode. Figure 2b shows the CV curves of the GO-CH-EDC/NHS-modified electrode in Zobel’s solution (0.001 mol L^−1^ Fe (CN)_6_^4−/3−^ solution containing 0.1 mol L^−1^ KCl) at different scan rates (0.02–0.2 V s^−1^ vs. Ag/AgCl). As seen, the peak currents were increased by increasing the scan rates, revealing excellent electroactivity of the as-prepared electrode.

### Optimization of sensing conditions

**Figure 3:**
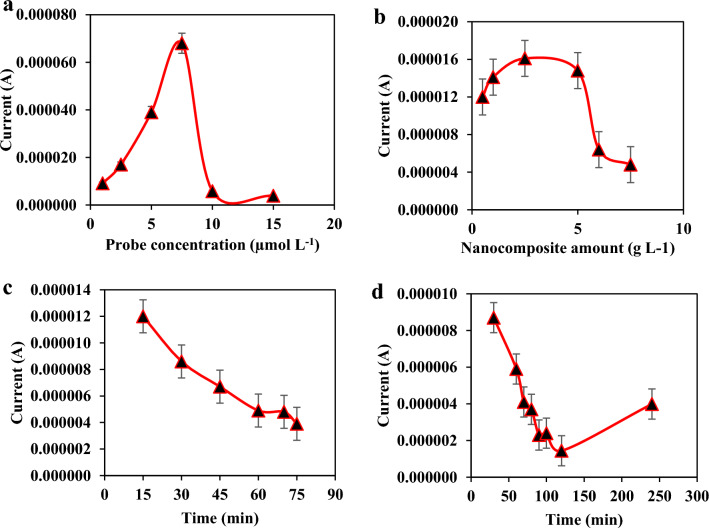
The effect of (**a**) the amount of DNA probe, (**b**) graphene oxide amount, (**c**) immobilization time of probe on the electrode, and (**d**) interaction time between bioelectrode and target DNA on the detection of MAP.

To reach the maximal sensitivity toward detection of MAP, the various affecting factors on the current intensity and interaction between the probe and target DNA, including the amount of probe, the immobilization time of ssDNA, the interaction time between the probe ssDNA and target DNA, and the concentration of nanocomposite were optimized (Fig. 3).The probe concentration was optimized between 1 and 15 µmol L^−1^, and the results were shown in Fig. 3a, revealed that the peak current reached its maximal value when 7.5 µmol L^−1^ of ssDNA was used to modify the GO-CH-DEC/NHS modified electrode. Besides, the effect of the GO content in the GO-CH composite on MAP detection was evaluated by changing its amount over 0.5–7.5 mg (Fig. 3b). The results showed that, up to 2.50 ± 0.01 mg of GO, the oxidation peak current was increased by increasing the GO content of the nanocomposite and then declined. It may be related to improving the surface area of the GO-CH composite by increasing the GO content, consequently enhancing the electron conductivity and increasing the peak current intensity. Moreover, the effect of the immobilization time of the ssDNA on the electrode surface was optimized over an immobilization time of 15.0–75.0 min, revealing that the sensitivity of the as-prepared nanobiosensor was maximized after the ssDNA immobilization within 70.0 min on the surface of the electrode (Fig. 3c). In addition, the effect of the interaction time between the probe ssDNA and target DNA on the sensor sensitivity was also checked upon an interaction time ranging from 30.0 to 240.0 min. The results are shown in Fig. 3d, demonstrating that the oxidation peak currents significantly decreased at 120.0 min and then leveled off. Hence, 120 min (2.0 h) was selected as the optimal interaction time between the probe ssDNA and target DNA.

### Linearity and detection limit

**Figure 4:**
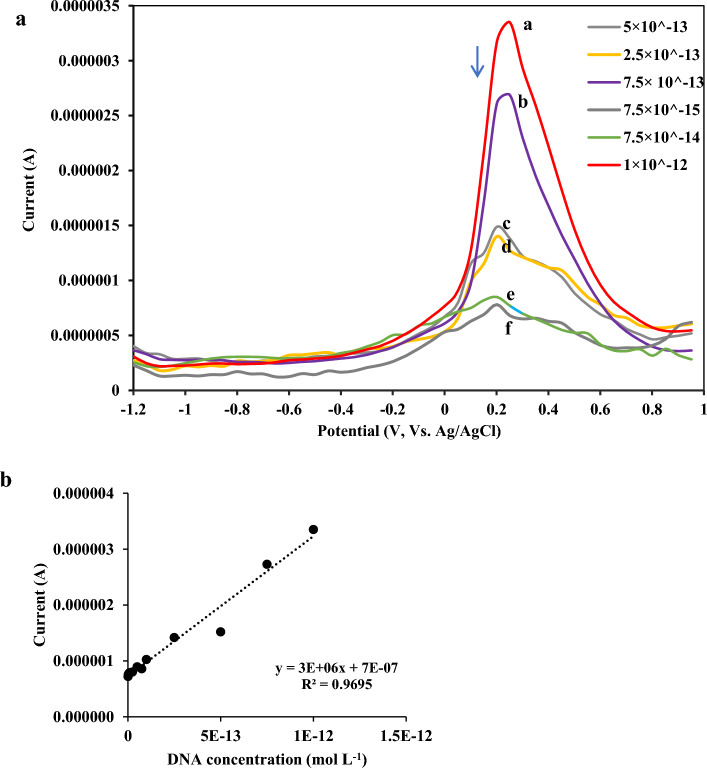
Analytical performance of the ssDNA-stabilized GO-CH-EDC/NHS modified bioelectrode: (**a**) DPVs of the electrochemical biosensor for target DNA detection at concentrations between (1.0 × 10^–15^ mol L^−1^ and 1.0 × 10^–12^ mol L^−1^) (**a**); 1.0 × 10^−12^ M (**b**); 7.5 × 10^−13^M (**c**); 5 × 10^−13^ M (**d**); 2.5 × 10^−13^M (**e**); 7.5 × 10^−14^ M (**f**); 7.5 × 10^−15^ M (**b**) The calibration plot of DPV peak current.

The developed ssDNA-stabilized GO-CH-EDC/NHS bioelectrode was utilized to determine different concentrations of the target DNA of MAP. The analytical responses were recorded by DPV analysis with different concentrations of the target DNA. The results shown in Fig. 4a revealed that the peak current intensity decreased significantly by increasing target DNA concentration, which may be associated with its phosphate backbone. Besides, a major net negative charge causes a further increase in the peak reduction. At optimal experimental conditions, a linear working range of 1.0 × 10^−15^–1.0 × 10^−12^ mol L^−1^ was obtained for MAP detection using the developed method (Fig. 4b). Besides, the detection limit of the as-prepared biosensor was calculated by 3s law (x̄_b_ + 3S_b_)^31^, providing a detection limit as low as 1.53E^−13^ mol L^−1^ for detecting the target DNA of MAP.

### Figures of merits

The figures of merits of the developed nanobiosensor, including repeatability, reproducibility, stability and specificity, were investigated.

**Figure 5:**
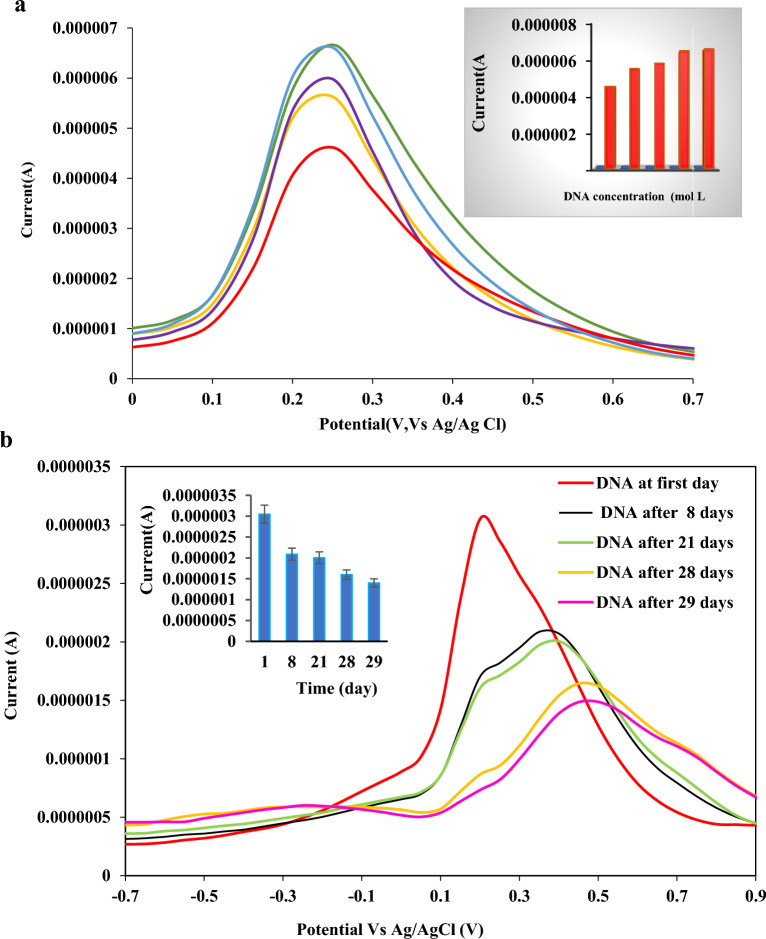
(**a**) Repeatability of the as-prepared bioelectrode with 1.0 × 10^–13^ mol L^−1^ target ssDNA and, Insert: histogram of electrochemical responses of biosensor with repeated fabrications (**b**) stability, Insert: changes in the peak current of DPVs recorded using the DNA biosensor after hybridization with 1.0 × 10^–13^ mol L^−1^ of DNA in consecutive days.

The reproducibility of the prepared nanobiosensor was evaluated by preparing the three different bioelectrodes. The process of hybridization/de-hybridization was repeated five times. Same concentration of target ssDNA (1 × 10^−13^) was used to hybridize the biosensor,DPVs were recorded after each repetition. After recording, the biosensor was de-hybridized by soaking it in hot water for 5 min at 95 °C. The results revealed that the difference between the response of the different bioelectrode toward quantification of MAP is negligible, and an %RSD as low as ~ 10% (n = 5) was obtained for the developed method, revealing admirable reproducibility of the proposed method. Regarding the repeatability studies, %RSD as low as 4.7% was obtained for 5 replicate measurements, indicating high repeatability of the results of the designed nanobiosensor (Fig. 5a). Besides, the stability of the as-prepared bioelectrode was investigated at room temperature within a long storage time of about three weeks (Fig. 5b). The results revealed that 65 and 49% of the analytical signal were saved after 21 days and 29 days of storage of the bioelectrode, exhibiting excellent stability of the developed bioelectrode. "The specificity of the biosensor for the detection MAP is a result of the careful design and optimization of the biosensor, as well as the use of appropriate controls and reference samples to ensure its accuracy and reliability.

### Selectivity

"It is well-known that a DNA-based biosensor has high selectivity toward its corresponding analyte if it can distinguish between matched and mismatched sequences^32,33^. Hence, the selectivity of the as-prepared bioelectrode toward discrimination of complementary, non-complementary, etc., was investigated by recording DPV responses at the same concentrations of 1.0 × 10^–13^ mol L^−1^.The DPVs before and after hybridization with target, complementary, and non-complementary ssDNA sequences are shown in Figure s4.

**Figure 6:**
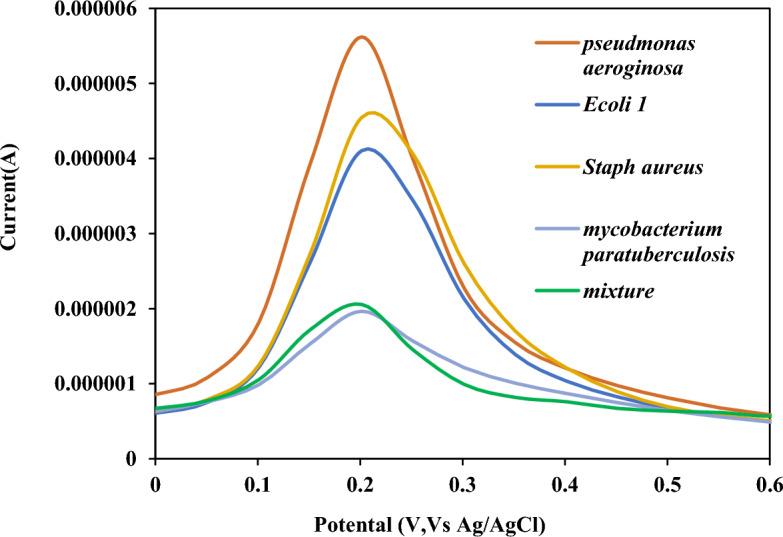
Specificity of the DNA biosensor: (**a**) specificity of the DNA biosensor, using four different PCR products (1.0 × 10^–13^ mol L^−1^) as interferences, and a mixture of the above four interferences and *MAP.*

The results showed that, on exposure to a complementary target sequence, a significant reduction can be observed in peak current, pointing to the strongest hybridization power of the complementary target. In contrast, the non-complementary sequence cannot result in a reduction of the peak current. The order of peak current decrement was as follows:

Target > 1-Basemismatched >  > Non-complementary, ssDNA sequences, revealing the excellent selectivity of the as-prepared bioelectrode. In addition, ssDNA-stabilized GO-CH-EDC/NHS bioelectrodes were designed for comparative conditions to evaluate the sensitivity of the biosensor. For this purpose, the PCR products of four bacterial samples (*E. coli, Staphylococcus aureus, Pseudomonas aeruginosa*, and a mixture of these) were assessed. As illustrated in (Fig. 6), the electrochemical characterization of the DNA biosensor was performed using differential pulse voltammetry (DPV). The DPV responses to the PCR products of the four interferences were compared to that of the blank sample to determine if the biosensor produced a significant electrochemical signal in response to these interferences. The results showed that the biosensor had excellent sensitivity for the detection of *M. paratuberculosis*, as it was able to distinguish the target DNA from other bacterial samples. The study also highlights the importance of appropriate controls and reference samples to ensure the biosensor's accuracy and reliability. Overall, the method involved evaluating the biosensor's ability to detect MAP specifically and accurately using electrochemical techniques and comparing the results to those of other bacterial samples.

### Real sample analysis

**Figure 7:**
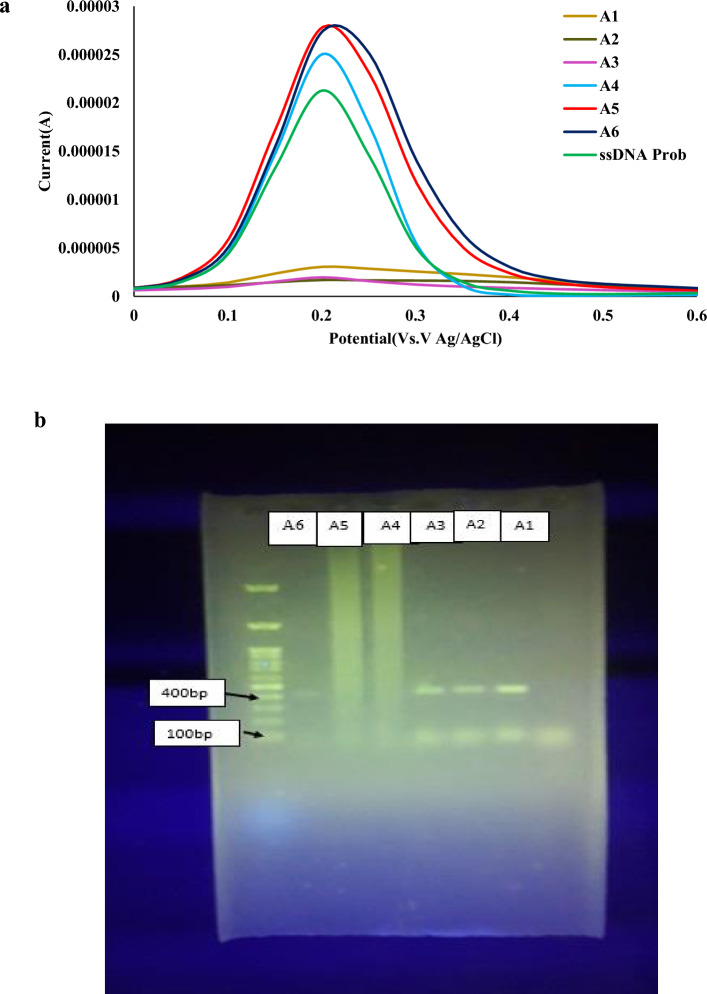
A comparative analysis with PCR: (**a**) DPV response for detecting in stool samples#A1-A6 with the same concentration of (1.0 × 10^–13^ mol L^−1^) using the developed biosensor and (**b**) the gel electrophoresis photographs of PCR (standard method) obtained from stool samples#A1-A6.

In this study, the stool samples were selected as real media for feasibility checking the as-prepared ssDNA-stabilized GO-CH-EDC/NHS modified bioelectrode toward MAP detection in natural media and point-of-care on-site digenesis of JD. To understand the applicability of biosensor and determine the real sample, we extracted the MAP DNA from six suspected stool specimens (Samples no.: #A1-A6), which was then analyzed by both the as-prepared bioelectrode and the standard polymerase chain reaction (PCR) followed by gel electrophoresis (Fig. 7). The results showed that samples no. #A1-A3 had the correct size, whereas samples no. #A4-A6 were negative in agarose gel electrophoresis. The agarose gel electrophoresis for the positive MAP sample revealed products at fragment sizes of 142 bp, as can be seen in Fig. 7b. Following this, the same sample was assessed using the fabricated DNA biosensor in a real-time setting, and the resulting current was compared, as depicted in Fig. 7a. Consequently, the DPV response demonstrated a decrease in the peak current using A1-A3 samples compared to the ssDNA probe. Conversely, the DPV response for A4-A6 samples resulted in an increased current response compared to A1-A3 samples, indicating a lack of hybridization between the DNA probe and PCR product. The findings revealed a significant DPV response, distinguishing a signal between positive and negative MAP, thereby demonstrating the potential of the fabricated electrochemical DNA sensor for selective and effective detection of MAP from the PCR products.

Notably, using the biosensor and obtaining the SD of blank, a sample was considered as positive (i.e., infected with MAP) when it produced a peak current lower than the Ip of the bare bioelectrode. The qualitative results for diagnosing John’s disease using the developed method compared to the results obtained from the standard form were shown in Table 1, revealing that the results of the as-prepared bioelectrode are in good agreement with those of the standard PCR method. Besides, quantitative experiments were performed to quantify MAP concentration in reals samples. The results shown in Table 2 revealed accurate and reliable results for quantification of MAP using the as-prepared bioelectrode.

**Table 1:** The qualitative results for diagnosis of John’s disease using the developed method compared to the results obtained from standard method.

Sample (stool) no	Standard method*	Developed nano-biosensor
A1	Positive**	Positive
A2	Positive	Positive
A3	Positive	Positive
A4	Negative	Negative
A5	Negative	Negative
A6	Negative	Negative

*PCR-gel electrophoresis.

**The term “positive” represents that the animal infected by MAP.

**Table 2:** Quantitative results of the MAP detection using the as-prepared bio-electrode.

Sample	Founded MAP concentration (nM)	RSD% (n = 3)
Stool	0.0035	22.2
Stool	0.0055	33.45
Stool	0.0050	19.2
Stool	0.0049	38.0
Stool	0.0173	24.0
Stool	0.0037	4.4

## Methods

### Materials and reagents

All materials used in this study were obtained in their analytical or biological grades. Deacetylated chitosan (CH, 75%), citric acid (CA), 1-ethyl-3-(3-dimethylaminopropyl) carbodiimide hydrochloride (EDC, 98%), N-hydroxysuccinimide (NHS, 98%), potassium hexacyanoferrate(III) (K_3_[Fe(CN)_6_]), sulfuric acid (H_2_SO_4_, 95.0–98.0%), KCl, were obtained from Merck Company. Milli-Q water was used for the preparation of all reagent solutions. The phosphate buffer solution with a pH over 2.0–8.0 was used as the working buffer solution, as reported^34^. The DNA probe was designed for the IS900 gene, specifically for MAP. All oligonucleotides were provided by Takapouist Biotech. Co. Ltd. (Iran). All oligonucleotide stock solutions were prepared with 10.0 mM in Milli-Q water (pH: 7.4) and stored at − 20 °C. Notably, the details of the oligonucleotide sequences used in this study are provided in Table s2.

### Instruments

A Potentiostat/galvanostat µAutolab® TypeIII (EcoChemie, Utrecht, Netherlands) controlled by NOVA 1.6 software in connection with a conventional three-electrode system composed of the reference electrode (Ag/AgCl, saturated KCl), a bio-modified GCE as the working electrode and a platinum wire as the counter electrode was utilized for electrochemical measurements, A Spectrum RXI (FT-IR) was used for recording the FT-IR spectra of the as-prepared materials. An Analytic Jena Flex cycler (Germany) was utilized for performing PCR standard assays. A Nano-Drop one/one^C^ microvolume UV–Visible spectrophotometer was used to determine DNA concentrations. A TESCAN-Vega3 scanning electron microscope was used for recording the SEM images.

### Materials synthesis and electrode preparation

#### Synthesis of graphene oxide nanoparticles

The graphene oxide nanoparticles were initially synthesized by direct heating solvent-free method based on the literature^35^. To do this, citric acid was used as an appropriate carbon source. In a typical synthesis, 5.00 g of citric acid (Merk, Germany) was introduced into a beaker, followed by direct heating at 200 °C for about 2.0 h to produce solid black graphene oxide nanoparticles. Notably, after 5.0 min of the reaction, a liquated yellow-colored product was produced. The color was changed to orange within 30.0 min. Finally, after 2 h from the solvent-free heating the orange liquid transferred into the final blackish solid graphene oxide nanoparticles.

#### Synthesis of graphene oxide-chitosan (GO-CH) nanocomposite

The GO-CH nanocomposite was prepared by directly mixing 5.0 mg graphene oxide nanoparticles into 2.0 mL of 0.05% (w/v) chitosan (Orbital, India) solution upon ultra-sonication for 10.0 min. The as-prepared nanocomposite was then used for modification of GCE^36^.

#### Fabrication of GO-CHI-EDC/NHS modified electrode

**Figure 8:**
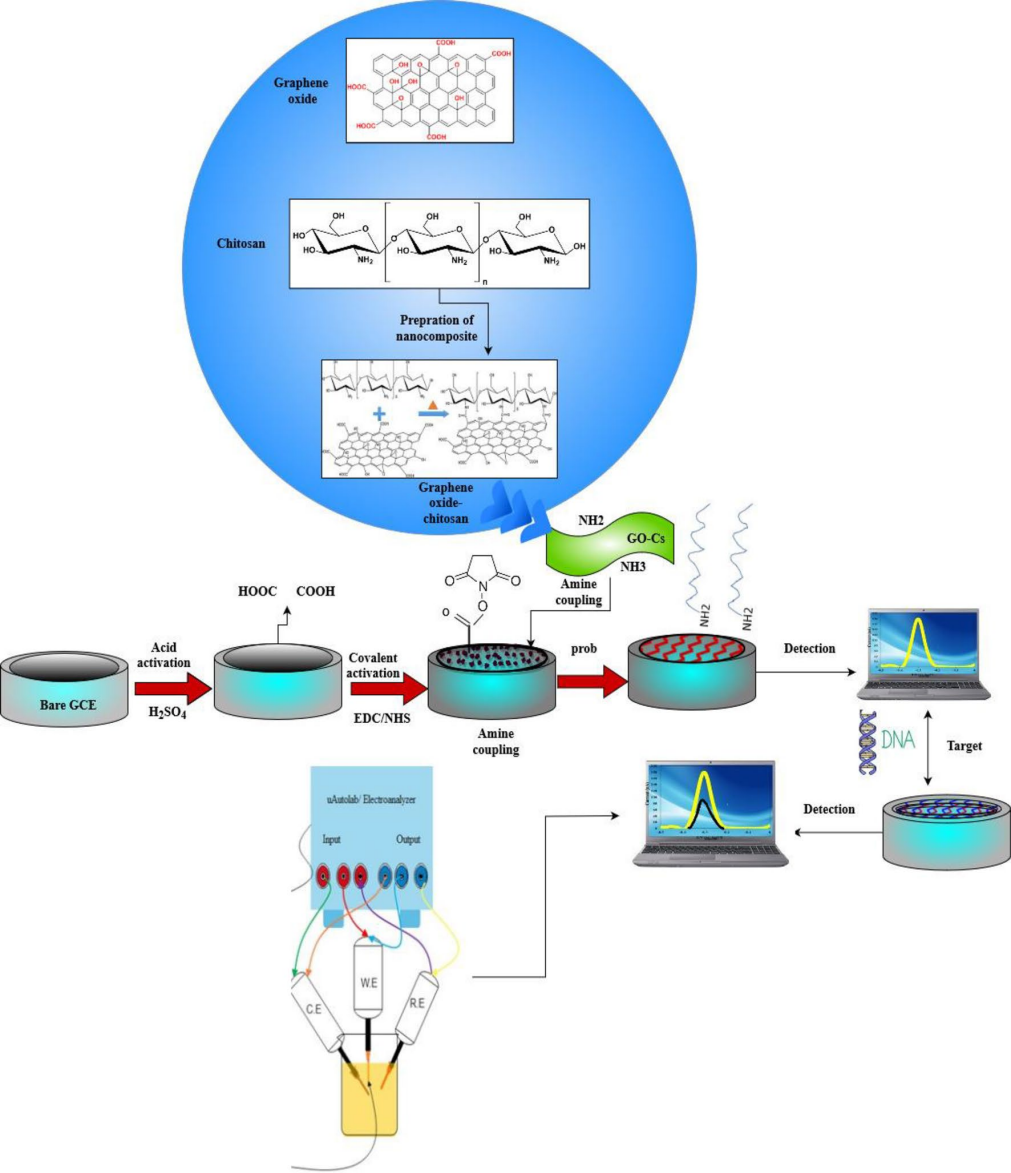
Schematic illustration of the stepwise electrochemical fabrication process: the preparation of graphene oxide nanocomposite (GO) by ultra-sonication in chitosan (CH) solution. The nanocomposite was attached with amine coupling reaction between the primary amine groups of CH and active N-hydroxy succinimide (NHS) esters anchored on the activated glassy carbon electrode (GCE) surface. The covalently-activated GCE surfaces were then modified by ss DNA.

To prepare the GO-CHI-EDC/NHS modified electrode, the GCE electrode was initially polished with Al2O3 powder (particle size of 1.0 mm) for 15.0 min, washed with pure water, and dried at ambient temperature. After that, the acid-activation of the electrode was performed into 0.5 M H_2_SO_4_ over a potential range of + 1.0 to − 1.0 V at a scan rate of 100.0 mV/s^−1^ for 30 sequential cycles. After that, the electrode was placed into a 1.5 mL EDC solution (50.0 m mol L^−1^) for 1.0 h, then dipping into 100.0 mM NHS solution for 2.0 h. The electrode was then dried under the nitrogen atmosphere. Afterward, the nanocomposite was prepared by mixing a desired amount of graphene oxide (5.0 mg) in 2.0 mL of CH and ultra-sonicated for 10.0 min, subsequently (10 µl) of prepared nanocomposite solution was dropped on the surface of the activated GCE followed by incubation at room temperature overnight, washing with Mili-Q water, and drying under the N_2_ atmosphere. The modification steps are illustrated in Fig. 8.

#### Fabrication of ssDNA-stabilized GO-CHI- EDC/NHS modified electrode

To fabricate the ssDNA-stabilized GO-CHI- EDC/NHS modified electrode, the commercial probe ssDNA was immobilized on the surface of the GO-CHI-EDC/NHS modified electrode by 10.0 μl ssDNA dropping on the electrode surface, followed by incubation at room temperature for 1.5 h. The electrode was then rinsed with MiliQ water to remove unabsorbed materials. After that, the hybridization detection was carried out through the dropping 10.0 μl of the hybridization solution (containing the target, 1-base mismatched, or non-complementary ssDNA sequences) and the complementary ssDNA (a concentration over 1.0 × 10^–18^–1.0 × 10^–7^ mol L^−1^) on the surface of the modified electrode. Notably, the hybridization process was performed at room temperature within 2.0 h, followed by biosensor washing with MiliQ water and drying under the N_2_ atmosphere. (Purity: 99.995%).

### Recommended procedure for MAP electrochemical biosensing

Electrochemical measurements were performed using a conventional three-electrode system by the ssDNA-stabilized GO-CH-EDC/NHS modified bioelectrode as the working electrode. The CVs of the potential range were taken between − 1.0 and 1.0 V at a scan rate of 100 mV/s^−1^. Besides, the DPV measurements were performed from − 1.2 to 1.0 V (vs. Ag/AgCl) at a step potential of 5.0 mV with a modulation amplitude of 0.025 V, a modulation time of 0.05 s, an interval time of 0.5 s, and a scan rate of 100.0 mV s^−1^. A mixed solution (pH: 7.4) of 0.1 mol L^−1^ KCl and 0.001 mol L^−1^ K_3_Fe (CN)_6_/K_4_Fe(CN)_6_ (mole ratio of 1:1) was employed as the redox marker. In addition, the stability of the developed biosensor was evaluated by recording its DPVs in different time intervals upon two weeks of storage at room temperature For checking the selectivity of the developed biosensor toward MAP detection, 1-base mismatched and non-complementary ssDNA sequences (1.0 × 10^–13^ mol L^−1^) was immobilized on the surface of the GO-CH-EDC/NHS modified electrode and the as-prepared bioelectrode was utilized for selectivity checking. Notably, the working electrode was placed in a 1.50 mL tube with 1.0 mL acetone. It was sonicated for 10.0 min, washed with isopropanol, and dried upon nitrogen purging.

### Practical application of the developed ssDNA-stabilized GO-CH-EDC/NHS-modified bioelectrode

Stool samples were selected as real media for feasibility checking the as-prepared ssDNA-stabilized GO-CH-EDC/NHS modified bioelectrode toward MAP detection in real media and point-of-care on-site digenesis of JD. In this regard, ten stool samples were collected from Kharameh farms. After that, the DNA of MAP was purified using a FavorPrep™ Stool DNA Isolation Mini Kit Taiwan according to the manufacturer’s guidelines. The extracted DNA samples were then stored at − 20 °C for future uses toward real sample analysis for both qualitative and quantitative detection of MAP using the as-prepared bioelectrode. It is notable that the PCR method was utilized as a standard method for proving the results of the developed process. To do the PCR assay, the genomic DNA was initially denatured at 94 °C for 5 min, followed by 30 cycles at 94 °C for 1.0 h, 59 °C for 1.0 h, 72 °C for 2 h, and 10 °C for 0.5 h. After the product was obtained, it was visualized using a 1.5% agarose gel and a UV–visible system (Lambda 365, Perkin Elmer, US).

## Conclusion

A novel, low-cost, high throughput, highly sensitive, and ultraselective electrochemical DNA nanobiosenor was developed to accurately detect MAP in real media for practical diagnosis of JD. The method was designed based on the immobilization of graphene oxide and chitosan biopolymer on the surface of the glassy carbon electrode, modified by electrochemical immobilization of GO and chitosan biopolymer, followed by activation of biopolymer via EDC/NHS coupling system. Afterward, the commercial probe DNA was stabilized on the activated electrode surface to prepare an ultra-selective nanobiosensor for MAP sensing.

The electrode was characterized by several characterization methods. The DNA hybridization between the nanobiosensor and target DNA was confirmed by cyclic voltammetry and differential pulse voltammetry. At optimal experimental conditions, a linear range over 1.0 × 10^–15^–1.0 × 10^–12^ mol L^−1^ a detection limit as low as 1.53 × 10^−13^ mol L^−1^, a repeatability (%RSD, n = 5) of 4.7%, and a reproducibility (%RSD, n = 5) of about 10%, along with ultra-selectivity and proper longtime stability was achieved for the developed nanobiosensor. Finally, it was employed for qualitative and quantitative analysis of real samples toward a practical diagnosis of JD, revealing highly accurate results. Considering the results as mentioned above, the developed nanobiosensor can be used for functional clinical JD diagnosis.

### Supplementary Information

Supplementary Information.

## Data Availability

Correspondence and material requests should be addressed to S. H.
