# Supplementary material for: Introducing a portable electrochemical biosensor for Mycobacterium avium subsp. paratuberculosis detection using graphene oxide and chitosan
Source: Sci Rep. 2024 Jan 2;14:34. doi: 10.1038/s41598-023-50706-z (PMC10761741; doi:10.1038/s41598-023-50706-z)
Supplement: Supplementary file 1 — Supplementary Information. [file 41598_2023_50706_MOESM1_ESM.docx]

**Supporting information**

**Introducing a portable electrochemical biosensor for *Mycobacterium* *avium* subsp. *paratuberculosis* detection using graphene oxide and chitosan**

Nahid Naghshgar^1^, Saied Hosseinzadeh*^2^, Abdollah Derakhshandeh^1^, Ruhollah Shaali^3^ Mohammad Mahdi Doroodmand^3^,

*^1^Department of Pathobiology, School of Veterinary Medicine, Shiraz University, Shiraz, Iran*

*^2^Department of Food Hygiene and Public Health, School of Veterinary Medicine, Shiraz University, Iran*

*^3^Department of Chemistry, College of Science, Shiraz University, Shiraz 71454, Iran*

**For correspondence:* [*hosseinzadeh@shirazu.ac.ir*](mailto:hosseinzadeh@shirazu.ac.ir) *(S. Hosseinzadeh)*

*
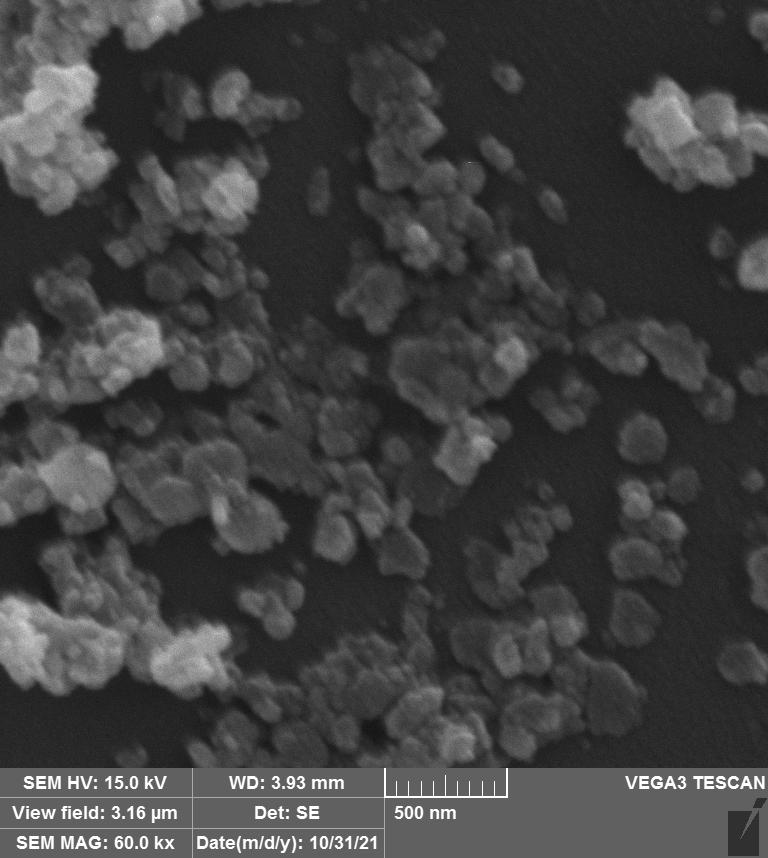
*

**Figure S1.** The SEM image of graphene oxide nanoparticles

**Figure S2.** The XRD pattern of graphene oxide nanoparticles

**Figure S3.** FT-IR spectra of GCE after modification, (a) glassy carbon electrode oxidation in sulfuric acid (b) GCE after EDC/NHS and chitosan bonding, (c) GCE after GO-CH bonding on the electrode surface, and (B) GO-CH-EDC/NHS modified GCE after probe and DNA binding on the electrode surface.

**Figure S4.** Selectivity of the prepared electrode: DPVs recorded using the biosensor before and after hybridization with target, 1-base mismatched, probe ,and non-complementary ssDNA sequences of 1.0 × 10^-13^ mol L-1.

**Table S1.** The EDX results of the bare GCE, EDC/NHS modified GCE, GO-CH-EDC/NHS modified GCE, and ssDNA-stabilzed GO-CH-EDC/NHS modified bio electrode electrode.

| Elements | W% of elements after oxidation of electrode surface | W% of elements after EDC/ NHS addition | W% of elements after CHI/GO reaction | W% of elements after Probe and DNA binding |
| --- | --- | --- | --- | --- |
| C | 83.89 | 92.74 | 65.29 | 75.48 |
| N | - | 5.30 | 12.77 | 15.44 |
| O | 14.63 | 1.89 | 19.32 | 10.46 |
| S | 1.48 | 0.07 | 0.02 | 0.01 |
| Total | 100 | 100.00 | 100.00 | 100.00 |
|  |  |  |  |  |

**Table S2.** Details of the IS900 gene, probes and primers used in this study.

| Amplicon | Nucleotide sequence 5′-3′ | Product length |  |  |
| --- | --- | --- | --- | --- |
| Forward primer  P90(413 bp) | 5′GAA GGG TGT TCG GGG CCG TCG CTT AGG3′ | 27 | (Millar et al. 1996) |  |
| Reverse primer p91(413 bp)  Prob sequence(ssDNA) | 5′GGC GTT GAG GTC GAT CGC CCA CGT GAC3′  5′ GAA GGG TGT TCG GGG CCG TCG CTT AGG 3′ | 27 | (Millar et al. 1996) |  |
| target ssDNA  (complementary) | 5′CCT AAG CGA CGG CCC CGA ACA CCC TTC3′ | 27 |  |  |
| 1-base mismatched ssDNA | 5′CCT AAG CCA CGG CCC CGA ACA CCC TTC3′ | 27 |  |  |
| Non complementary ssDNA | 5′AAG CCT ATG TTT AAT ATC CAC TTA CCA 3′ | 27 |  |  |
|  |  |  |  |  |
